# Hybridization in palms (Arecaceae)

**DOI:** 10.1002/ece3.70014

**Published:** 2024-07-14

**Authors:** Christine D. Bacon, Adrian Hill

**Affiliations:** ^1^ Department of Biological and Environmental Sciences University of Gothenburg Gothenburg Sweden; ^2^ Gothenburg Global Biodiversity Centre Gothenburg Sweden

**Keywords:** Arecaceae, biome, evolution, hybridization, islands, macroecology, palm

## Abstract

Hybridization has significant evolutionary consequences across the Tree of Life. The process of hybridization has played a major role in plant evolution and has contributed to species richness and trait variation. Since morphological traits are partially a product of their environment, there may be a link between hybridization and ecology. Plant hybrid species richness is noted to be higher in harsh environments, and we explore this hypothesis with a keystone tropical plant lineage, palms (Arecaceae). Leveraging a recent literature review of naturally occurring palm hybrids, we developed a method to calculate hybrid frequency, and then tested if there is phylogenetic signal of hybrids using a phylogeny of all palms. Further, we used phylogenetic comparative methods to examine the interaction between hybrid frequency and presence in dry environments, on islands, and the species richness of genera. Phylogenetic generalized least squares models had stronger support than models of random association, indicating phylogenetic signal for the presence of hybrids in dry and island environments. However, all *p*‐values were >.05 and therefore the correlation was poor between hybridization and the trait frequencies examined. Presence in particular environments are not strongly correlated to hybrid frequency, but phylogenetic signal suggests a role in its distribution in different habitats. Hybridization in palms is not evenly distributed across subfamilies, tribes, subtribes yet plays an important role in palm diversity, nonetheless. Increasing our understanding hybridization in this economically and culturally important plant family is essential, particularly since rates are projected to increase with climate change, reconfiguring the dynamics and distribution of biodiversity.

## Graphical abstract

Across the plant Tree of Life, hybridization has contributed to the formation of species complexes and biased our understanding of taxonomic diversity and phylogenetic relationship, palms included. To increase understanding, we explore hybridization in palms and test for ecological correlates of hybridization. We reveal that hybridization is not evenly distributed and our results suggest that hybridization influences palm diversity and distribution.

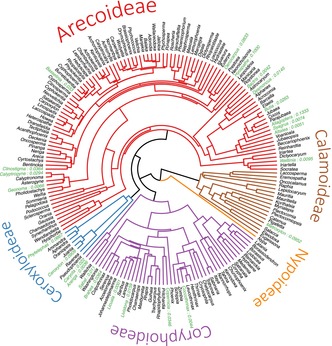

## 1 INTRODUCTION

Hybridization is the result of sexual reproduction between species, usually congeneric species or subspecific taxa, but can occur between species from different genera (Stebbins, 1950). In natural populations, hybridization can act in opposition to divergence, introduce adaptive variation into a population, drive the evolution of stronger reproductive barriers, or generate new lineages (Goulet et al., 2017). Hybridization may occur as isolated occurrences with little to no evolutionary consequences (Grant, 1981), but may also generate high evolutionary novelty (e.g. allelic, structural, and phenotypic variation; Rieseberg et al., 2003). Repeated hybridization through introgression can cause alleles of one species to be incorporated into another. Sometimes large populations of hybrids, or hybrid zones, can form between two adjacent or co‐distributed species. The processes of introgression and formation of hybrid zones has played a major role in the evolution of several plant families (Rieseberg & Wendel, 1993). Hybridization has strong phylogenetic signal, where it occurs more frequently in certain plant families and genera than others (Schley et al., 2021).

Hybrids are offspring of two different species and can be studied from the perspective of the mechanisms of reproductive isolation, including speciation and species concepts (e.g. Harrison & Larson, 2014; Nolte & Tautz, 2010), from a taxonomic perspective (e.g. Funk, 1985; Rothfels, 2021), or from the perspective of hybridization as an evolutionary process (e.g. Soltis & Soltis, 2009). Here, we explore hybridization in the palm family (Arecaceae) from these perspectives. Palms are a taxonomically (ca. 2600 species; Baker & Dransfield, 2016) and functionally diverse clade (Kissling et al., 2019) that are characteristic of tropical ecosystems (Couvreur et al., 2011). Palms have rich phylogenetic (Baker et al., 2009; Faurby et al., 2016), trait (Kissling et al., 2019), and distribution data (GBIF; https://www.gbif.org) available as well. Despite this rich knowledge of palm diversity, hybridization is poorly studied (Barrett, McKain, et al. 2019), and further, hybridization is a relatively unexplored area of evolutionary biology in tropical floras (Schley et al., 2021).

Recent review of hybridization in the Neotropics suggested that because dry environments (e.g. savanna, grassland) are relatively young in comparison to wet ones (e.g. rainforest, montane), hybridization is widespread in these floras (Schley et al., 2021). Because hybridization is associated with the colonization of new environments (Stebbins, 1985), we hypothesize that dry environments have more hybrid palm species. In addition, dispersal and adaptation to novel environments renders homoploid hybrid speciation more likely, without the necessity for instantaneous reproductive isolation as in polyploid hybrid speciation (Coyne & Orr, 2004). Since hybrid speciation is most likely to occur in island scenarios (Kerbs et al., 2017), we hypothesize that islands also have higher proportions of hybrid palm species. Lastly, because a higher number of congeners could increase the potential for hybridization, we treated the number of species per genus as a trait to ask whether hybridization is more common in larger genera. We test these hypotheses by developing a calculation for hybrid frequency per genus in palms and use phylogenetic comparative methods (Freckleton et al., 2002). We discuss our results in light of the implications on systematics studies and methods to detect hybridization. Understanding hybridization in an economically and culturally important group such as palms is fundamental, since rates are projected to increase with climate change, which will alter the dynamics and distribution of biodiversity (Vallejo‐Marín & Hiscock, 2016).

## 2 MATERIALS AND METHODS

A recent compilation of data on naturally occurring palm hybrids (Henderson, 2022) was used to test for ecological correlated of hybridization. Numbers of species per genus were taken from Govaerts et al. (2020) or from the most recent monographs. Hybrids between infraspecific taxa and those in cultivated palms were not included since they are not directly related to this work.

The species list of naturally occurring palm hybrids was used to infer the number of potential hybrids per genus. We calculated potential hybrids per genus as GR × (GR − 1)/2, where GR is Genus Richness, or the number of species in the genus. Hybrid frequency for each genus was then calculated as the number of actual hybrids / potential hybrids. All data handling and analysis was performed in R v. 4.1.2 (R Core Team, 2014).

To test our hypotheses regarding the interaction between certain traits and hybridization, we referred to data available in the literature. We used the species coded as wet (2200 species), dry (291 species), and generalist (48 species) from Cássia‐Silva et al. (2019) to test our hypothesis that the proportion of potential hybrids in dry environments is higher. To test our hypothesis that the proportion of potential hybrids is higher in island environments, we used the species coded as mainland (1400 species) and island (1150 species, 822 from continental and 328 from volcanic islands) from Cássia‐Silva et al. (2020); any species found on both the mainland and islands were excluded.

An updated version (Hill et al., 2023) of the palm species‐level phylogeny of Faurby et al. (2016) was trimmed to the genus level phylogeny by removing all except for a random, single tip per genus. Fractions of species with specific states for each trait was calculated per genus. We used phylogenetic comparative methods to ask if, when we account for autocorrelation due to phylogenetic relatedness, is there a correlation between the hybrid frequency and a trait? Phylogenetic generalized least squares (PGLS) models were used to leverage knowledge of phylogenetic relationships to produce an estimate of expected covariance in our data (Freckleton et al., 2002). Closely related species are assumed to have more similar traits because of their shared ancestry and hence produce more similar residuals from the least squares regression line. By accounting for the expected covariance structure of these residuals, modified slope and intercept estimates are generated that can account for interspecific autocorrelation due to phylogeny. PGLS models were constructed using the “pgls” function in caper v. 1.0.1. (Orme et al., 2018).

For the PGLS model hybrid frequency per genus was regressed against the frequency of each trait per genus. The PGLS model was compared to a white noise model using the Akaike information criterion (AIC; Burham & Anderson, 2002) in geiger v. 2.0.10. (Harmon et al., 2008). White noise models were constructed using the “fitContinuous” function in geiger and assumes no phylogenetic signal, meaning that traits are not autocorrelated due to phylogenetic relatedness. When AIC is lower (indicating better model fit) for the white noise models, it means the traits are randomly distributed and when AIC is lower for PGLS models, it indicates the trait is influenced by phylogeny (Harmon et al., 2008).

## 3 RESULTS

**FIGURE 1:**
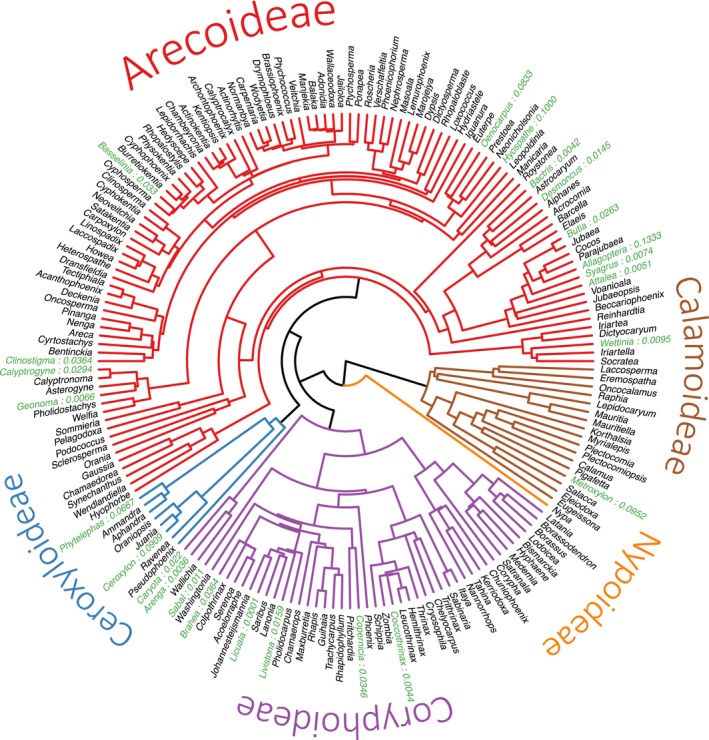
Naturally occurring palm hybrids, marked in green with their estimated hybrid frequency listed next to the genus name, plotted on an updated version (Hill et al., 2023) of the palm species‐level phylogeny of Faurby et al. (2016) that was trimmed to the genus level. Subfamilies are marked in colors and names on the outer portion of the circular phylogeny.

One hundred one naturally occurring palm hybrids were identified in Henderson (2022). Although hybrids are found throughout the palm phylogeny, they are not evenly distributed across the tree (Figure 1).

### 3.1 Phylogenetic generalized least squares

Habitat type had three possible trait states: (1) wet or (2) dry habitat specialist, or (3) habitat generalist. For all the three habitat trait states, the AIC was significantly lower for the PGLS models than the white noise models, indicating better model fit for the PGLS models than the white noise models. This means that correcting for the effect of phylogenetic relationships produce better fitting models, indicating that phylogeny influences covariation between hybrid frequency and habitat type. The ΔAIC values (PGLS AIC – white noise AIC) were (1) wet: −974.7, (2) dry: −962.40, and (3) generalist: −573.5, respectively.

Results for all the PGLS models were found to not be significant based on the *p*‐value (all *p*‐values were greater than *p* = .05):

1. Wet habitat specialist: residual standard error = 0.001837 on 163 degrees of freedom, multiple *R* ^2^ = .002719, *F*‐statistic = 0.4444 on 1 and 163 df, and *p*‐value = .506.
2. Dry habitat specialist: residual standard error = 0.001839 on 163 degrees of freedom, multiple *R* ^2^ = .0002438, *F*‐statistic = 0.03975 on 1 and 163 df, and *p*‐value = .8422.
3. Habitat generalist: residual standard error = 0.001824 on 163 degrees of freedom, multiple *R* ^2^ = .017, *F*‐statistic = 2.818 on 1 and 163 df, and *p*‐value = .09511.

Land type had two possible trait states: (1) mainland or (2) island. Being a binary trait, the two states share the same results. The PGLS model AIC was lower than the white noise model AIC. The ΔAIC value was −1055.08, meaning that the PGLS model fit is significantly better than white noise model fit.

The PGLS results for land type (one set of results a for binary state) were: residual standard error = 0.001823 on 163 degrees of freedom, multiple *R* ^2^ = .01777, adjusted *R* ^2^ = .01175, *F*‐statistic = 2.95 on 1 and 163 df, *p*‐value = .0878.

Genus richness was the final examined trait, referring to the total number of species in any given palm genus. Models were made to test correlation between genus richness and hybrid frequency to see whether larger genera have more hybrid species. The ΔAIC for the genus richness models was −2557.44, which indicated significantly better fit of the PGLS than the white noise model.

The PGLS results for genus richness were: residual standard error = 0.001839 on 163 degrees of freedom, multiple *R* ^2^ = .0003795, adjusted *R* ^2^ = −.005753, *F*‐statistic = 0.06189 on 1 and 163 df, and *p*‐value = .8039.

## 4 DISCUSSION

Hybridization is increasingly recognized as an important evolutionary process in plants as it is fundamental to speciation, extinction, dispersal (through range expansion and invasion), and allows for increased trait diversity in both natural and agricultural systems. Here we used palms to test whether hybrids are more common (1) in dry environments, (2) on islands, and (3) in larger genera. We scored species for these traits and leveraged a phylogeny of palms in a macroevolutionary framework. We found that the traits are influenced by phylogeny, meaning they have phylogenetic signal, although the statistical significance of the results is low. We use our results to review current understanding and the implications of hybridization in palms.

### 4.1 Macroecological analyses of hybrids and their correlations with traits

All white noise models had higher (worse) AIC score than PGLS models, meaning PGLS models are better than random and present phylogenetic signal. However, all *p*‐values were >.05, meaning there was no meaningful correlation between hybrid frequency per genus and the trait frequencies in question. So, for example, hybrids are more common in dry environments, but the correlation is not statistically significant. Further evidence of an interaction of hybridization and dry‐adapted palms was shown by increased genome sizes, as understood from certain repetitive elements, in arid environments (Schley et al., 2022).

Other studies examining correlates of hybridization detected several associations between hybridization rates and plant traits such as perenniality, woodiness, outcrossing rate, pollination syndrome, reproductive systems, genome size, and genome size variation (Mitchell et al., 2019). Traits such as woodiness and outcrossing rates likely co‐vary with traits examined here, such as presence or absence on islands (Carlquist, 1974). Hybridization is common within palm genera found on Hawai'i and Cuba (Bacon, Baker, et al., 2012; Bacon, McKenna, et al., 2012) and may be related to soil characteristics. This pattern has been closely examined in insular *Howea* palm species, where ancestral hybridization led to eventual speciation (Osborne et al., 2019). Certain traits may correlate more strongly with the formation of a hybrid, and others with the persistence of a hybrid (Mitchell et al., 2019). While not differentiated here, different environmental settings appear crucial to the presence of naturally occurring palm hybrids.

We also examined whether the size of a genus affects hybrid frequency, but the correlation is weak. While the reason for a lack of correlation is not clear, the use of Linnean ranks as a unit or framework of comparison may be problematic. The overwhelming majority of hybridization occurs within genera, meaning that larger genera have a larger potential for hybridization events. However, this assumes that hybridization is as likely in larger genera as in smaller genera. It is ultimately genetic differentiation between species that determines whether hybridization is possible. Because Linnean ranks are arbitrary, there is no guarantee that congeners in larger genera are equally differentiated and therefore likely to hybridize as congeners in smaller genera. Further, genera are a product of humans dividing the world up into perceived natural breaks at a certain level of morphological complexity, so there may be some arbitrariness and conflicts in the breakpoints used for recognition of a clade as a genus, and therefore in the comparability of genera. Lastly, the use of hybrid frequency may degrade the power to detect phylogenetic signal in the traits of interest.

### 4.2 The extent and taxonomic distribution of hybridization in palms

There are 182 currently recognized genera of palms. Naturally occurring hybrids have been reported in 19 genera (10% of the total; Henderson, 2022). However, of these 182 genera, 54 are monospecific and about 40 have few, allopatric species. This leaves about 90 genera having two or more species, some of which are distributed sympatrically, in which hybrids could potentially occur. Hybrids are reported in 21% of these genera.

There are ca. 2473 currently recognized species of palms, yet only 111 naturally occurring hybrids have been reported (Henderson, 2022). Because several of these hybrids have the same parent, only about 130 palm species (5% of the total) are involved in hybridization. Within genera it appears that the most widespread and variable species are more likely to form hybrids. Examples include *Attalea maripa* and *A. speciosa*, *Bactris acanthocarpa* and *B. major*, *Copernicia hospida*, *Desmoncus polyacanthos*, *Geonoma deversa*, *Livistona australis*, *Oenocarpus bacaba*, *Sabal palmetto*, and *Syagrus coronata* and *S. romanzoffiana*. As shown from the phylogenetic comparative results, larger genera do not significantly have more hybrids.

Hybrids are intuitively related to the domestication process. While some palms may have been at least semi‐domesticated by Indigenous groups (e.g. *Bactic gasipaes*, *Euterpe precatoria*, *Mauritia flexuosa*; Levis et al., 2017) there is a clear history of domestication in one of the oldest tree crop species in the world, the date palms (*Phoenix*; Flowers et al., 2019). *Phoenix* species are interfertile and crossing distinct species leads to fertile hybrid offspring (Gros‐Balthazard, 2013). Introgression of relevant functional genes from wild relatives into cultivated varieties of dates is important to breeders (Pérez‐Escobar et al., 2021) and although deeply fascinating, falls outside the scope of the work here on naturally‐occurring hybrids.

### 4.3 The implications of hybridization for palm systematics

At both the generic and specific level there appear to be rather few hybrids in palms, although they are certainly under‐reported. Most of the hybrids discussed here are reported by systematists who are revising genera based on herbarium specimens. Different systematists have different propensities to recognize hybrids. Hybrids are anyway difficult to detect from specimens, and they are usually hypothesized based on sympatry of putative parents and morphological intermediacy of putative hybrids.

Furthermore, the sample size of most species in herbaria is extremely small compared with population size, and thus few hybrid specimens may be present in herbaria. Most reported hybrids (about 70%) appear to be isolated occurrences and may have little significance for systematics. About 20 hybrids are relatively widespread, and seven hybrid zones have been reported (Henderson, 2022). Sometimes hybrids are problematic for systematics. For example, *Washingtonia* has been treated as having either one or two, sympatric or allopatric species. Recent studies using molecular data have shown that even in this small, well‐studied genus variation is complex and difficult to unravel (Klimova et al., 2018; Villanueva‐Almanza et al., 2021).

Hybridization may play a prominent role in formation of species complexes. Gene flow and hybridization can have either a destructive or creative effect on the evolution of species complexes (Arnold, 1992). Differential introgression in palm species complexes is exemplified by potential hybrids in *Geonoma*, present both between species and different morphotypes of *G. brongniartii*, *G. camana*, *G. deversa*, *G. elegans*, *G. leptospadix*, *G. longivaginata*, *G. maxima*, *G. macrostachys*, *G. orbignyana*, *G. pauciflora*, *G. pinnatifrons*, *G. poeppigiana*, *G. pohliana*, *G. schottiana*, *G. stricta*, and *G. undata* (Bacon et al., 2021; Henderson, 2011). Deeper genomic and population‐level sampling within the genus is needed to understand the hybrid origin of the species complexes in *Geonoma* (Loiseau et al., 2019). Another example of hybridization leading to the formation of species complexes is found in *Pritchardia martii* and co‐distributed congeners on Hawai'i (Bacon, McKenna, et al., 2012). The tendency for island colonizers to fill niches quickly leads to species that are ecologically but not genetically isolated, often leading to hybridization (Carlquist, 1974) and subsequently, the formation of species complexes.

For phylogenetic studies, several palm studies have indicated that hybrids may have influenced results (e.g. Bacon et al., 2021; Bacon, Baker, et al., 2012; Barrett, Sinn, et al., 2019; Loiseau et al., 2019). Taken that phylogenetic trees can only present dichotomously diverging branches, they cannot be accurate if the analysis includes hybrids (McDade, 1990). It has even been suggested that if hybridization is indeed common in plants, then phylogenetics (at least methods that do not account for gene flow in the model) should not be applied to plants at all (Hull, 1979). Using a series of parental and F_1_ hybrids, McDade (1992) suggested that hybrids do not erode phylogenetic structure unless they are between distantly related parents. More recently, using the multispecies coalescent model and inter‐ and intra‐specific sampling it was shown that even a small amount of gene flow can have a large impact on the genetic history of species (Jiao & Yang, 2021). Advances in genomics and computational biology has led to new approaches to infer phylogenetic trees in the face of gene flow and the inclusion of hybrid taxa (e.g. Xu, 2000).

### 4.4 Detecting hybridization

Hybridization was traditionally inferred using hybrid indices derived from data of individuals from putative hybrid populations based on the similarity to phenotypes of parental forms (Grant, 1981). These tests largely require growing plants, either in field experiments, greenhouse, or growth chamber. Determining potential hybrids in the field can be difficult without controlled or other experimental approaches, and certainly required observation and collection of multiple individuals per population across the hybrid zone. Further, it is difficult to detect hybridization in the herbarium, first because it is hard to detect from specimens and secondly, since the sample size in most herbaria is much smaller than their natural population size (Henderson, 2022). Genomic data, on the other hand, provide a window into the process and there are a variety of genomic‐based approaches to identifying hybrids.

As described above, hybrids cause non‐dichotomous branching in phylogenies and therefore can be visualized in different ways to find them in the sample. For example, phylogenetic networks display hybridization by nodes connecting hybridizing species in a phylogenetic tree. Pérez‐Escobar et al. (2021) used this approach to determine how a hybrid *Phoenix* species related to other species in the genus based on observed genetic distance between sequences, with no correction for multiple substitutions (p‐distance, SplitsTree software; Huson, 1998). Visual identification of hybrids can also be done by mapping gene trees on top of each other, where trees that agree in topology and branch lengths are consistent and those that are not appear as webs, indicating hybridization (DensiTree software; Bouckaert, 2010). Bacon, McKenna et al. (2012) implemented this method to examine admixture in *Pritchardia*, finding evidence for hybridization in certain clades of the tree. These approaches can be particularly useful for inferring the timing, magnitude, and direction of gene flow (e.g. Degnan, 2018; Schliep et al., 2017; Solís‐Lemus & Ané, 2016; Than et al., 2008).

Hybrids can also be detected through population assignment tests, that identify global (genome‐average) and local (locus‐specific) ancestry from population level sampling. STRUCTURE uses a hierarchical Bayesian model to identify populations and estimate global ancestry for each sampled individual based on allele frequencies (Pritchard et al., 2000) and has been extended to estimate locus‐specific ancestry (Lawson et al., 2018). Using population assignment tests in *Washingtonia* palms, Villanueva‐Almanza et al. (2021) tested for the presence of a hybrid zone, and found highly structured, non‐admixed populations. For less computationally intensive estimates of genetic ancestry, Bayesian (fastSTRUCTURE; Raj et al., 2014) and Likelihood‐based (ADMIXTURE; Alexander et al., 2009) programs can be particularly useful for large datasets.

Several phylogenomic analyses have been developed to infer introgression, for example using *D*‐statistics (ABBA‐BABA statistic) and f4‐ratios (Patterson et al., 2012). The ABBA‐BABA test is based on counts of ancestral (A) and derived (B) alleles in sets of four samples with known phylogenetic relationships (i.e. three ingroups and an outgroup). Two allele patterns, ABBA and BABA, are incongruent with the species tree BBAA and can be used to infer introgression. Under incomplete lineage sorting, the two patterns should be equally frequent; therefore, a significant excess of one pattern over the other (as evaluated with Patterson's *D* statistic) is indicative of introgression (Malinksy et al., 2021). Another approach is a Bayesian algorithm that calculates the proportion of alleles inherited from parental reference (hybrid index; Bailey, 2024). These analyses were combined to determine that *Ceroxylon* species benefit from gained variation from introgression via enhancement of population sizes by recreating a common genetic pool (Carvalho‐Madrigal & Sanín, 2024). This is an area of active growth and as the generation of larger datasets increases, is expected to increase our understanding of hybridization across all plants.

## 5 CONCLUSIONS

The processes of hybridization and introgression have significant evolutionary consequences and have played a major role in the evolution of several plant families. Across the plant Tree of Life, hybridization has contributed to the formation of species complexes and biased our understanding of the taxonomic diversity and phylogenetic relationship, palms included. There are about 2473 currently recognized species of palms, but only 111 naturally occurring hybrids have been reported. Within genera it appears that the most widespread and variable species are more likely to form hybrids, but the species richness of genera is not correlated to hybrid frequency. Presence in particular environments is also not strongly correlated to hybrid frequency. Our systematic review of hybridization in palms reveals that it is not evenly distributed across subfamilies, tribes, subtribes. Taken together, we find that hybridization plays a role in palm diversity and distribution, which is only more recently coming to light, but more data is needed. Understanding hybridization in this economically and culturally important plant family is essential, particularly since rates are projected to increase with climate change, reconfiguring the dynamics and distribution of biodiversity (Vallejo‐Marín & Hiscock, 2016).

## AUTHOR CONTRIBUTIONS

**Christine D. Bacon:** Conceptualization (equal); funding acquisition (equal); supervision (equal); writing – original draft (equal); writing – review and editing (equal). **Adrian Hill:** Data curation (equal); formal analysis (equal); methodology (equal); visualization (equal); writing – review and editing (equal).

### OPEN RESEARCH BADGES

This article has earned an Open Data badge for making publicly available the digitally‐shareable data necessary to reproduce the reported results (Appendix S1).

## Supporting information


Appendix S1

## Data Availability

The data supporting our findings are found Henderson (2022).
