# Supplementary material for: Hybridization in palms (Arecaceae)
Source: Ecol Evol. 2024 Jul 14;14(7):e70014. doi: 10.1002/ece3.70014 (PMC11246834; doi:10.1002/ece3.70014)
Supplement: Supplementary file 1 — Appendix S1 [file ECE3-14-e70014-s001.pdf]

Appendix 1. Naturally occurring palm hybrids.

Genus	Hybrid	Subfamily	Tribe	Subtribe	Reference
Basselinia	*B. gracilis × B. pancheri*	Arecoideae	Areceae	Basseliniinae	Pintaud 2006
Basselinia	*B. pancheri × B. deplanchei*	Arecoideae	Areceae	Basseliniinae	Pintaud 2006
Clinostigma	*C. samoense × C. warburgii*	Arecoideae	Areceae	unplaced	Hodel 2007b
Allagoptera	*A. campestris × A. leucocalyx*	Arecoideae	Cocoseae	Attaleinae	Moraes 1996
Attalea	*A. funifera × A. humilis (A. × voeksii)*	Arecoideae	Cocoseae	Attaleinae	Henderson 2020b
Attalea	*A. funifera × A. oleifera (A. piassabossu)*	Arecoideae	Cocoseae	Attaleinae	Henderson 2020b
Attalea	*A. humilis × A. oleifera (A. salvadorensis)*	Arecoideae	Cocoseae	Attaleinae	Henderson 2020b
Attalea	*A. maripa × A. sp. (A. cryptanthera)*	Arecoideae	Cocoseae	Attaleinae	Henderson 2020b
Attalea	*A. maripa × ?A. guianensis (A. macropetala)*	Arecoideae	Cocoseae	Attaleinae	Henderson 2020b
Attalea	*A. maripa × A. guianensis (A. camopiensis)*	Arecoideae	Cocoseae	Attaleinae	Henderson 2020b
Attalea	*A. maripa × A. guianensis (A. degranvillei)*	Arecoideae	Cocoseae	Attaleinae	Henderson 2020b
Attalea	*A. nucifera × A. butyracea*	Arecoideae	Cocoseae	Attaleinae	Henderson 2020b
Attalea	*A. speciosa ×A. eichleri (A. × teixeriana)*	Arecoideae	Cocoseae	Attaleinae	Bondar 1954
Attalea	*A. speciosa × A. compta (× Attabignya minarum)*	Arecoideae	Cocoseae	Attaleinae	Balick et al. 1987a
Attalea	*A. sagotii × A. barreirensis*	Arecoideae	Cocoseae	Attaleinae	Henderson 2020b
Attalea	*A. speciosa × A. maripa (A. dahlgreniana)*	Arecoideae	Cocoseae	Attaleinae	Henderson 2020b
Bactris	*B. acanthocarpa × B. oligoclada (B. × moorei)*	Arecoideae	Cocoseae	Bactridinae	Wessels Boer 1971
Bactris	*B. acanthocarpa × B. sp.*	Arecoideae	Cocoseae	Bactridinae	Henderson 2000
Bactris	*B. barronis × B. gasipaes*	Arecoideae	Cocoseae	Bactridinae	Henderson 2000
Bactris	*B. caudata × B. coloradonis*	Arecoideae	Cocoseae	Bactridinae	Henderson 2000
Bactris	*B. corossilla × B. maraja*	Arecoideae	Cocoseae	Bactridinae	Henderson 2000
Bactris	*B. incommoda*	Arecoideae	Cocoseae	Bactridinae	Henderson 2000
Bactris	*B. major × B. brongniartii*	Arecoideae	Cocoseae	Bactridinae	Henderson 2000
Bactris	*B. major × B. setulosa*	Arecoideae	Cocoseae	Bactridinae	Henderson 2000
Bactris	*B. mexicana × B. gracilior*	Arecoideae	Cocoseae	Bactridinae	Henderson 2000
Bactris	*B. setiflora × B. corossilla*	Arecoideae	Cocoseae	Bactridinae	Henderson 2000
Butia	*B. paraguayensis × B. lallemantii*	Arecoideae	Cocoseae	Attaleinae	Gaiero et al. 2011
Butia	*B. paraguayensis × B. yatay*	Arecoideae	Cocoseae	Attaleinae	Noblick 2014
Butia/Syagrus	*B. lallemantii × S. romanzoffiana*	Arecoideae	Cocoseae	Attaleinae	Brussa & Grela 2007
Butia/Syagrus	*B. eriospatha ×S. romanzoffiana (× Butyagrus paranaënsis)*	Arecoideae	Cocoseae	Attaleinae	Engels et al. 2021
Butia/Syagrus	*B. capitata × S. romanzoffiana (×Butyagrus nabonnandii)*	Arecoideae	Cocoseae	Attaleinae	Brussa & Grela 2007
Desmoncus	*D. leptoclonos × D. sp.*	Arecoideae	Cocoseae	Bactridinae	Henderson 2011
Desmoncus	*D. parvulus × D. sp.*	Arecoideae	Cocoseae	Bactridinae	Henderson 2011
Desmoncus	*D. polyacanthos × D. horridus*	Arecoideae	Cocoseae	Bactridinae	Henderson 2011
Desmoncus	*D. polyacanthos × D. mitis*	Arecoideae	Cocoseae	Bactridinae	Henderson 2011
Desmoncus	*D. polyacanthos × D. pumilus*	Arecoideae	Cocoseae	Bactridinae	Henderson 2011
Syagrus	*S. coronota × S. vagans*	Arecoideae	Cocoseae	Attaleinae	Noblick 2017

Syagrus	*S. comosa* × *S. elata*	Arecoideae	Cocoseae	Attaleinae	Noblick 2017
Syagrus	*S. coronota* × *S. microphylla*	Arecoideae	Cocoseae	Attaleinae	Noblick 2017
Syagrus	*S. glaucescens* × *S. oleracea*	Arecoideae	Cocoseae	Attaleinae	Noblick 2017
Syagrus	*S. pleioclada* × *S. duartei*	Arecoideae	Cocoseae	Attaleinae	Noblick 2017
Syagrus	*S. pleioclada* × *S. glaucescens*	Arecoideae	Cocoseae	Attaleinae	Noblick 2017
Syagrus	*S. romanzoffiana* × *S. glaucescens*	Arecoideae	Cocoseae	Attaleinae	Noblick 2017
Syagrus	*S. romanzoffiana* × *S. loefgrenii*	Arecoideae	Cocoseae	Attaleinae	Noblick 2017
Syagrus	*S. coronata* × *S. botryophora*	Arecoideae	Cocoseae	Attaleinae	Noblick 2017
Syagrus	*S. coronata* × *S. cearensis*	Arecoideae	Cocoseae	Attaleinae	Noblick 2017
Syagrus	*S. coronata* × *S. romanzoffiana*	Arecoideae	Cocoseae	Attaleinae	Noblick 2017
Syagrus	*S. romanzoffiana* × *S. flexuosa*	Arecoideae	Cocoseae	Attaleinae	Noblick 2017
Syagrus	*S. romanzoffiana* × *S. oleracea*	Arecoideae	Cocoseae	Attaleinae	Noblick 2017
Syagrus	*S. coronota* × *S. schizophylla*	Arecoideae	Cocoseae	Attaleinae	Noblick 2017
Hyospathe	*H. elegans* × *H. pittieri*	Arecoideae	Euterpeae		Henderson 2004
Oenocarpus	*O. bacaba* × *O. minor (O.* × *andersonii)*	Arecoideae	Euterpeae		Balick 1991
Oenocarpus	*O. bataua* × *O. bacaba*	Arecoideae	Euterpeae		Balick 1988
Oenocarpus	*O. bataua* × *O. mapora*	Arecoideae	Euterpeae		Henderson et al. 1995
Calyptrogyne	*C. brachystachys* × *C. ghiesbreghtiana*	Arecoideae	Geonomateae		Henderson 2005
Calyptrogyne	*C. brachystachys* × *C. trichostachys*	Arecoideae	Geonomateae		Henderson 2005
Calyptrogyne	*C. panamensis* × *C. fortunensis*	Arecoideae	Geonomateae		Henderson 2005
Geonoma	*G. deversa* × *G. leptospadix*	Arecoideae	Geonomateae		Henderson 2011b
Geonoma	*G. orbignyana* × *G. undata*	Arecoideae	Geonomateae		Henderson 2011b
Geonoma	*G. pauciflora* × *G. elegans*	Arecoideae	Geonomateae		Henderson 2011b
Geonoma	*G. poeppigiana* × *G. brongniartii*	Arecoideae	Geonomateae		Henderson 2011b
Geonoma	*G. camana* × *G. macrostachys*	Arecoideae	Geonomateae		Henderson 2011b
Geonoma	*G. deversa* × *G. brongniartii*	Arecoideae	Geonomateae		Henderson 2011b
Geonoma	*G. deversa* × *G. longivaginata*	Arecoideae	Geonomateae		Henderson 2011b
Geonoma	*G. interrupta* × *G. pinnatifrons*	Arecoideae	Geonomateae		Henderson 2011b
Geonoma	*G. pauciflora* × *G. pohliana*	Arecoideae	Geonomateae		Henderson 2011b
Geonoma	*G. pauciflora* × *G. schottiana*	Arecoideae	Geonomateae		Henderson 2011b
Geonoma	*G. poeppigiana* × *G. sp.*	Arecoideae	Geonomateae		Henderson 2011b
Geonoma	*G. undata* × *G. trigona*	Arecoideae	Geonomateae		Henderson 2011b
Geonoma	*G. deversa* × *G. occidentalis*	Arecoideae	Geonomateae		Henderson 2011b
Wettinia	*W. quinaria* × *W. kalbreyeri (W. oxycarpa)*	Arecoideae	Iriarteeae		Galeano & Bernal 2010
Calamus	*C. leloi* × *C. sp.*	Calamoideae	Calameae	Calaminae	Henderson 2020
Calamus	*C. insignis* × *C. longiusculus*	Calamoideae	Calameae	Calaminae	Henderson 2020
Calamus	*C. javensis* × *C. tenompokensis*	Calamoideae	Calameae	Calaminae	Henderson 2020
Calamus	*C. bacularis* × *C. myriacanthus*	Calamoideae	Calameae	Calaminae	Henderson 2020
Metroxylon	*M. paulcoxii* × *M. warburgii*	Calamoideae	Calameae	Metroxylinae	McClatchey 1998

Ceroxylon	*C. parvifrons* × *C. quindiuense*	Ceroxyloideae	Ceroxyleae		Sanín pers. comm.
Ceroxylon	*C. quindiuense* × *C. alpinum*	Ceroxyloideae	Ceroxyleae		Sanín pers. comm.
Ceroxylon	*C. peruvianum* × *C. echinulatum*	Ceroxyloideae	Ceroxyleae		Sanín pers. comm.
Ceroxylon	*C. quindiuense* × *C. vogelianum*	Ceroxyloideae	Ceroxyleae		Sanín pers. comm.
Phytelephas	*P. seemannii* × *P. macrocarpa*	Ceroxyloideae	Phytelepheae		Barfod 1991
Arenga	*A. westerhoutii* × *A. pinnata*	Coryphoideae	Caryoteae		Whitmore 1998
Caryota	*C. cumingii* × *C. rumphiana*	Coryphoideae	Caryoteae		Hahn & Sytsma 1999
Caryota	*C. monostachya* × *C. bacsosnensis*	Coryphoideae	Caryoteae		Hamh & Sytsma 1999
Caryota	*C. cumingii* × *C. mitis*	Coryphoideae	Caryoteae		Hahn & Sytsma 1999
Coccothrinax	*C. crinita* × *C. miraguama*	Coryphoideae	Cryosophileae		Suárez Oropesa 2015
Coccothrinax	*C. macroglossa* × *C. pseudorigida*	Coryphoideae	Cryosophileae		Craft 2017
Coccothrinax	*C. pauciramosa* × *C. orientalis*	Coryphoideae	Cryosophileae		Craft 2017
Coccothrinax/Th	*Coccothrinax* × *Thrinax*	Coryphoideae	Cryosophileae		Nauman 1989, 1990
Sabal	*S. minor* × *S. palmetto (× brazoriensis)*	Coryphoideae	Sabaleae		Goldman *et al.* 2011
Sabal	*S. minor* × *S. mexicana*	Coryphoideae	Sabaleae		Goldman et al. 2011
Sabal	*S. minor* × *S. palmetto (S. miamensis)*	Coryphoideae	Sabaleae		Wunderlin 1998
Brahea	*B. dulcis* × *B. nitida*	Coryphoideae	Trachycarpeae	unplaced	Ramírez-Rodríguez et al. 2011
Copernicia	*C. baileyana* × *C. yarey (C. curbeloi)*	Coryphoideae	Trachycarpeae	unplaced	Craft 2017
Copernicia	*C. cowellii* × *C. macroglossa (C. × dahlgreniana)*	Coryphoideae	Trachycarpeae	unplaced	Verdecia Pérez 2016
Copernicia	*C. hospita* × *C. brittonorum (C. occidentalis)*	Coryphoideae	Trachycarpeae	unplaced	Dahlgren & Glassman 1963
Copernicia	*C. hospita* × *C. cowellii (C. × shaferi)*	Coryphoideae	Trachycarpeae	unplaced	Dahlgren & Glassman 1963
Copernicia	*C. rigida* × *C. baileyana (C. molinetti)*	Coryphoideae	Trachycarpeae	unplaced	Craft 2017
Copernicia	*C. hospita* × *C. baileyana (C. × textilis)*	Coryphoideae	Trachycarpeae	unplaced	Dahlgren & Glassman 1963
Copernicia	*C. hospita* × *C. macroglossa (C. burretiana)*	Coryphoideae	Trachycarpeae	unplaced	Dahlgren & Glassman 1963
Copernicia	*C. hospita* × *C. rigida (C. sueroana)*	Coryphoideae	Trachycarpeae	unplaced	Dahlgren & Glassman 1963
Copernicia	*C. rigida* × *C. gigas (C. × vespertilionum)*	Coryphoideae	Trachycarpeae	unplaced	Dahlgren & Glassman 1963
Copernicia	*C. rigida* × *C. sp. (C. longiglossa)*	Coryphoideae	Trachycarpeae	unplaced	Craft 2017
Copernicia	*C. rigida* × *C. baileyana (C. oxycalyx)*	Coryphoideae	Trachycarpeae	unplaced	Craft 2017
Licuala	*L. paludosa* × *L. bruneiana*	Coryphoideae	Trachycarpeae	Livistoninae	Miyamoto et al. 2006
Livistona	*L. australis* × *L. decora*	Coryphoideae	Trachycarpeae	Livistoninae	Dowe 2009
Livistona	*L. australis* × *L. humilis*	Coryphoideae	Trachycarpeae	Livistoninae	Tucker 1975
Livistona	*L. humilis* × *L. inermis*	Coryphoideae	Trachycarpeae	Livistoninae	Rodd 1998
Livistona	*L. kimberleyana* × *L. lorophylla*	Coryphoideae	Trachycarpeae	Livistoninae	Rodd 1998
Livistona	*L. kimberleyana* × *L. mariae*	Coryphoideae	Trachycarpeae	Livistoninae	Rodd 1998
Livistona	*L. saribus* × *L. jenkinsiana*	Coryphoideae	Trachycarpeae	Livistoninae	Henderson & Nguyễn Quốc Dựng 2019
